# Endoscopic submucosal dissection for a giant circumferential early rectal cancer with massive schistosoma egg deposition: a case report

**DOI:** 10.3389/fsurg.2026.1888367

**Published:** 2026-08-06

**Authors:** Yuhang Cui, Linzhen Li, Yan Zhang

**Affiliations:** Department of Gastroenterology, Yijishan Hospital, Wannan Medical College, Wuhu, China

**Keywords:** colorectal adenomatous polyps, colorectal cancers, endoscopic submucosal dissection, fibrosis, schistosomiasis

## Abstract

Colorectal adenomatous polyps are well-established as the principal precancerous lesions underlying the adenoma-carcinoma sequence in colorectal carcinogenesis. The World Endoscopy Organization (WEO) consensus guidelines strongly advocate *en bloc* resection over piecemeal resection for laterally spreading lesions measuring >20 mm in diameter, given their elevated risk of superficial submucosal invasion. Nevertheless, achieving complete *en bloc* resection via endoscopic submucosal dissection (ESD) for circumferential lesions situated immediately adjacent to the anal verge and exceeding 10 cm in maximum dimension represents a formidable technical challenge. Herein, we report the successful *en bloc* ESD resection of a giant circumferential mixed-nodular laterally spreading lesion extending from the anal verge to 8 cm proximally, with postoperative histopathology confirming moderately differentiated adenocarcinoma (Figure 5). The patient presented with concomitant schistosomal colopathy, which further compounded the procedural complexity of ESD. Notwithstanding these challenges, meticulous perioperative assessment, refined surgical technique, and comprehensive postoperative management collectively demonstrate that ESD remains a safe and effective therapeutic modality for appropriately selected patients.

## Introduction

Colorectal polyps represent all protuberant excrescences projecting into the intestinal lumen, histopathologically categorized as either adenomatous or non-adenomatous subtypes. The overwhelming majority of colorectal cancers (CRC) evolve through the adenoma-carcinoma sequence over a protracted timeframe; consequently, endoscopic detection and eradication of adenomatous polyps during colonoscopic surveillance substantially reduces both CRC incidence and disease-specific mortality (1, 2). Among adenomatous subtypes, tubulovillous adenomas constitute the most prevalent histology (61%), with tubular and villous adenomas accounting for 29% and 11% of cases, respectively (3). Early detection and timely therapeutic intervention for CRC dramatically improve survival outcomes, with 5-year relative survival rates approaching 90% for localized disease (2).

For colorectal laterally spreading tumors (LSTs) ≥20 mm in diameter, the WEO international consensus emphasizes that *en bloc* resection should be the preferred approach instead of piecemeal resection for lesions at high risk of superficial submucosal invasion (≤1000 um) in the rectum, such as granular mixed-type LSTs (4). A clinical study found that the incidence of submucosal invasion in rectal LSTs reached 17.8%, further corroborating the clinical imperative underlying this consensus recommendation (5). Despite these guidelines, complete endoscopic submucosal dissection (ESD) for large, circumferential, mixed-type rectal LSTs with superficial submucosal invasion remains an extraordinarily challenging endoscopic endeavor.

This report describes a clinically challenging case of a large, circumferential rectal LST measuring >10 cm in maximum diameter, situated immediately adjacent to the anal verge with histologically confirmed superficial submucosal invasion. Compounding this complexity, the patient exhibited concomitant schistosomal colopathy, which induced severe intestinal wall fibrosis and further escalated the technical demands of the ESD procedure.

## Case report

**Figure 1:**
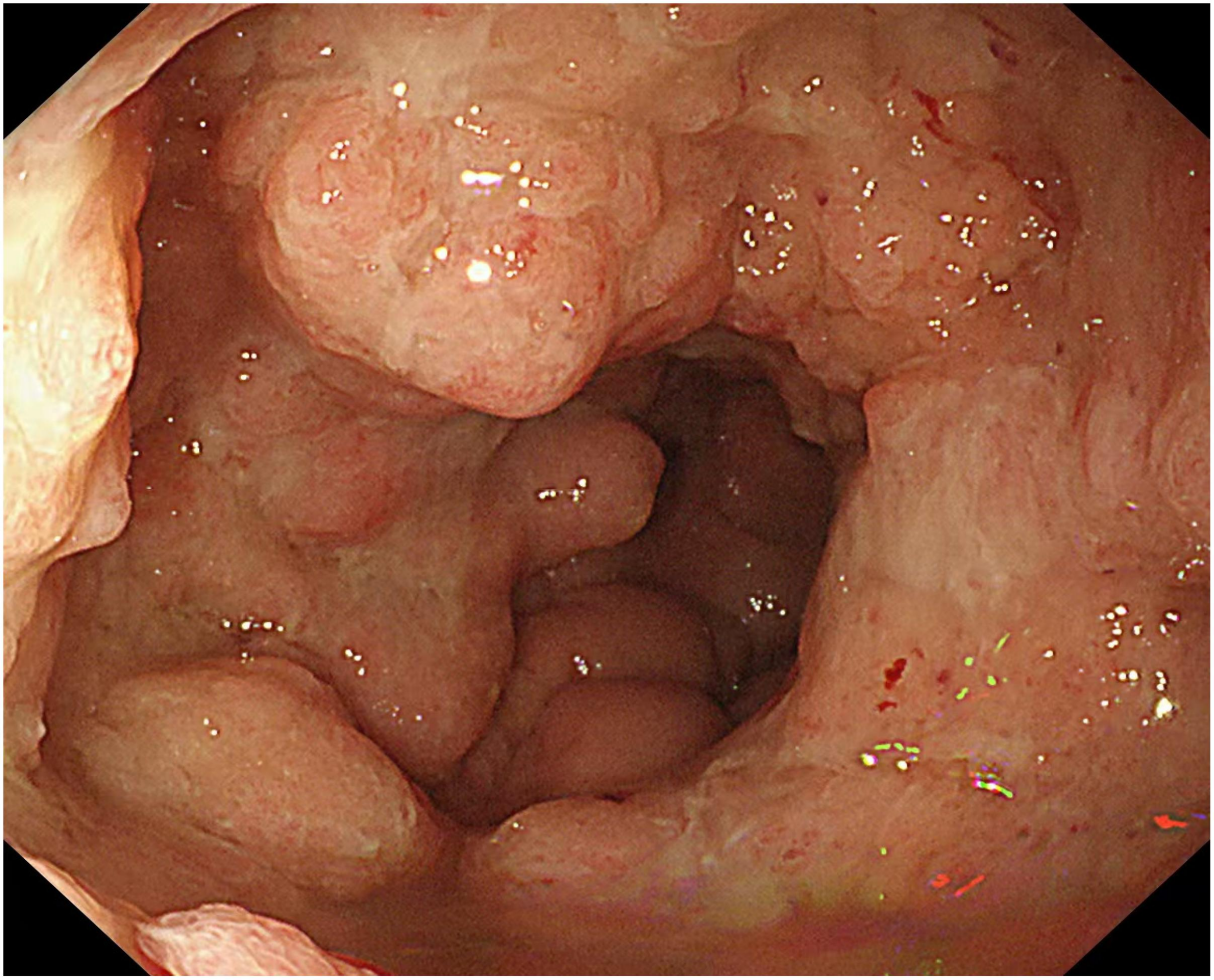
Colonoscopy revealed a giant circumferential mixed-nodular laterally spreading lesion extending from the anal verge to 8 cm above it.

A 77-year-old male patient presented to our outpatient department on December 29, 2025, with a 2-year history of progressive increased bowel frequency. Preprocedural colonoscopic evaluation revealed a giant circumferential rectal lesion, with initial biopsy histopathology confirmed a villoglandular adenoma (Figure 1).

Comprehensive preoperative lesion characterization was performed: employing the Paris classification system, the lesion was categorized as predominantly type 0-Is with focal 0-IIa components. Chromoendoscopy with magnification demonstrated primarily Kudo type IV pit patterns, with focal type V₁ pits and areas exhibiting V_N pit pattern disappearance indicative of malignant transformation. Narrow-band imaging (NBI) magnification revealed predominantly JNET type 2B vascular features across most of the lesion, with focal regions demonstrating JNET type 3 characteristics consistent with invasive carcinoma.

**Figure 2:**
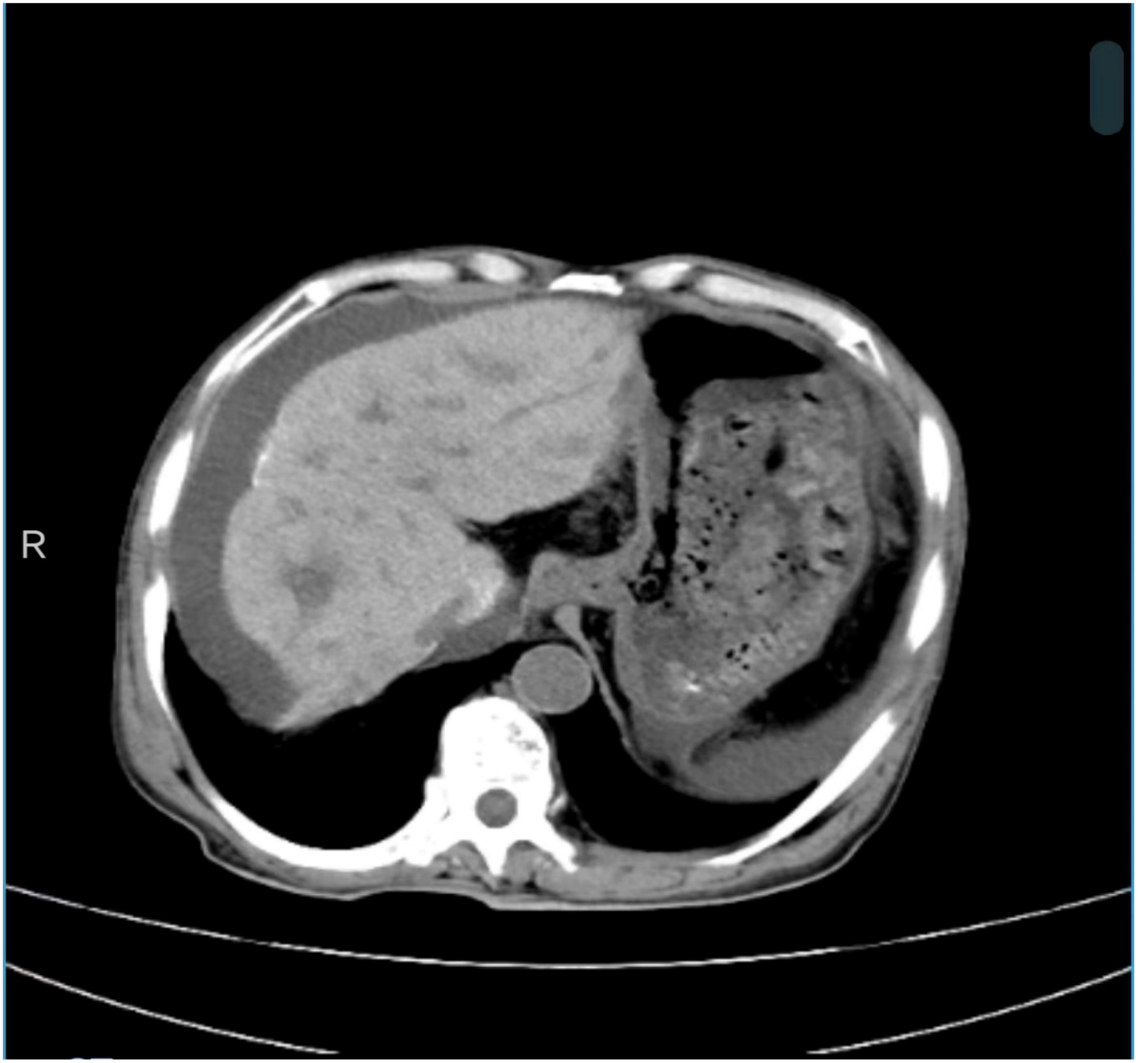
High-density calcification shadows can be seen in the patient's liver.

Given the lesion's substantial dimensions and circumferential configuration, ESD was deemed extremely difficult, and surgical resection was initially recommended. However, given the lesion's proximity to the anal verge, surgical resection would have necessitated abdominoperineal resection with permanent colostomy, precluding anal preservation. Therefore, the patient was admitted to our department to attempt ESD. Routine preoperative laboratory investigations were unremarkable apart from mild monocytosis. Abdominal computed tomography (CT) revealed high-density calcification shadows can be seen in the patient's liver (Figure 2). Upon targeted clinical inquiry, the patient disclosed a remote history of schistosomiasis infection acquired during prolonged residence in an endemic riverside region.

**Figure 3:**
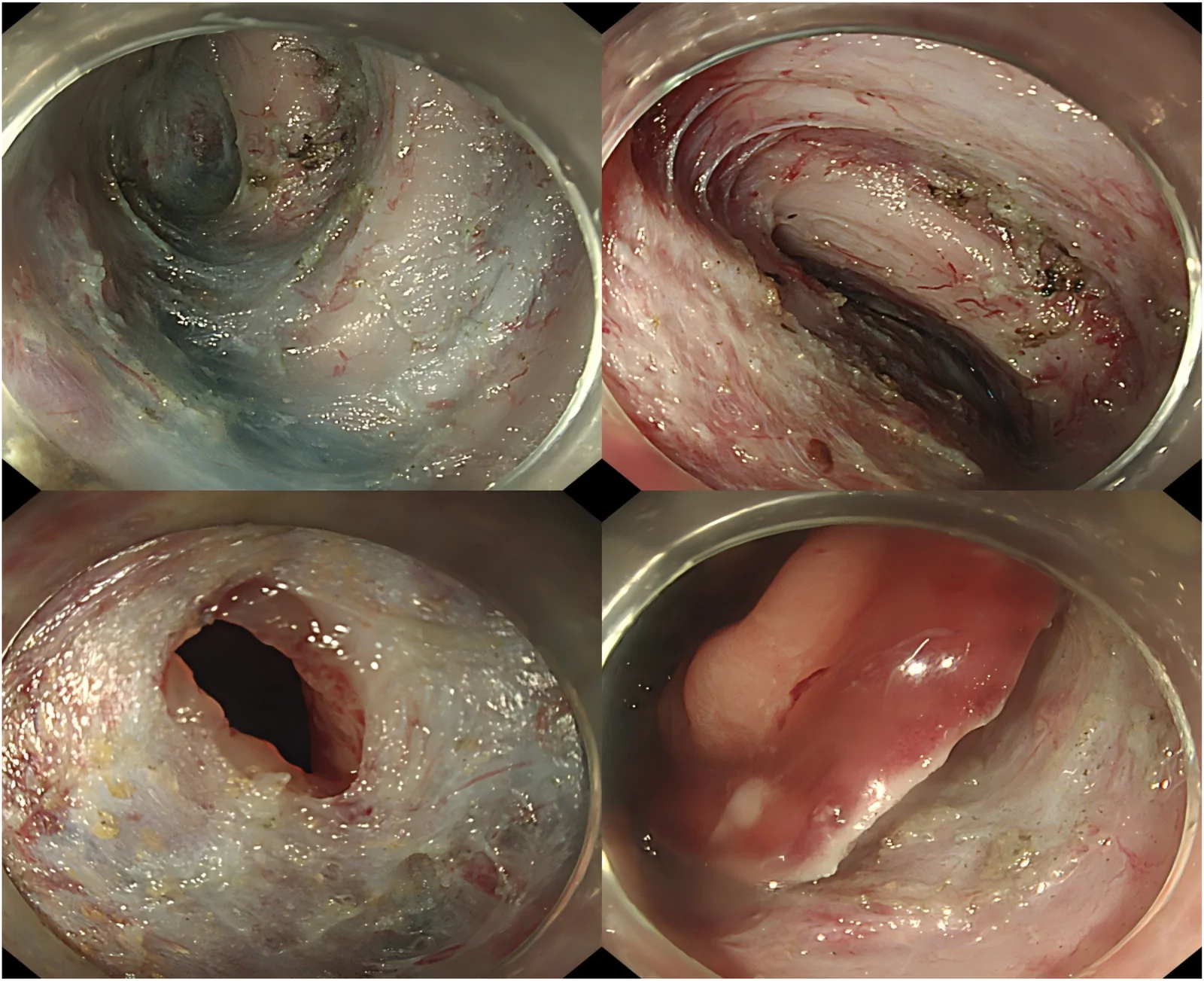
Intraoperative findings.

**Figure 4:**
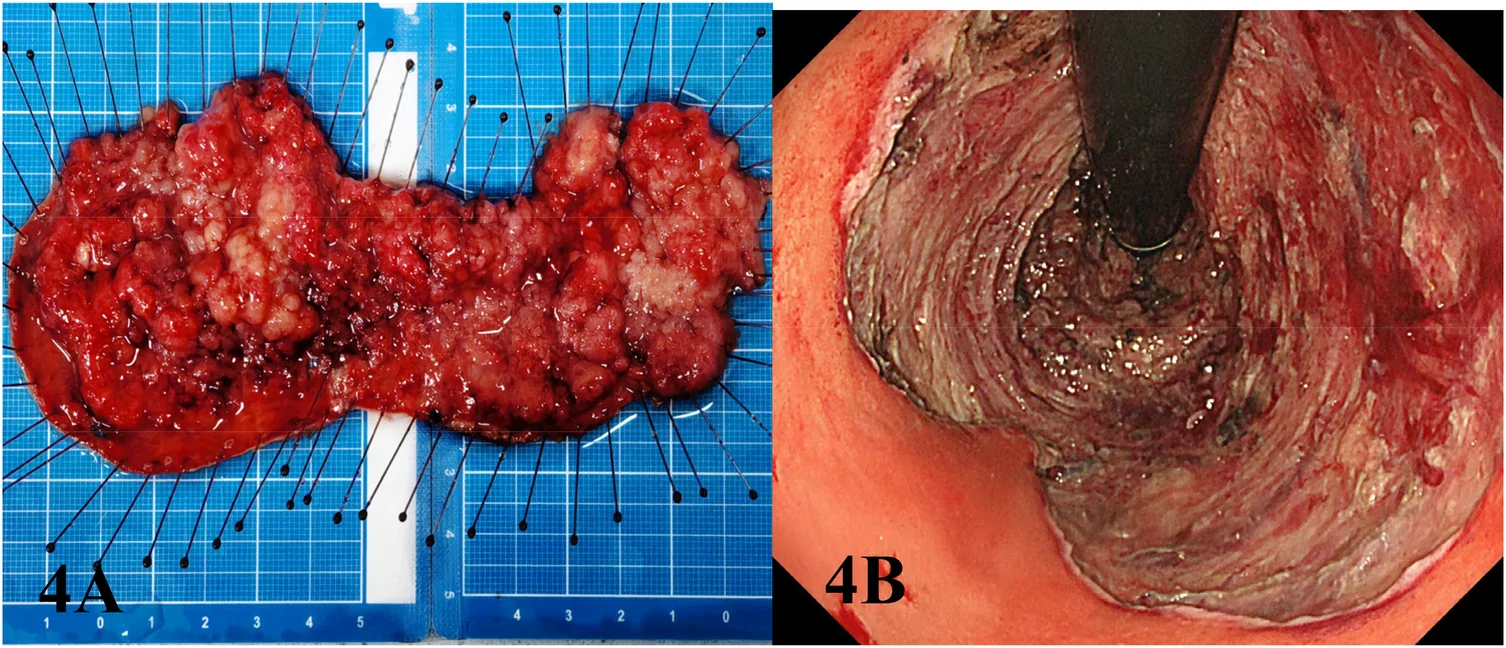
**(A)** the resected specimen measured approximately 14.5 cm × 6.5 cm after complete *en bloc* removal. **(B)** The wound bed after complete resection of the lesion showed no significant bleeding or perforation.

The patient underwent ESD of the rectum on January 7, 2026, under general anesthesia with the patient positioned in the left lateral decubitus position. Intraoperative endoscopy revealed a large circumferential nodular mixed-type LST extending from the anal verge to 8 cm above the anal verge. The procedure commenced with submucosal injection of normal saline supplemented with indigo carmine and epinephrine to facilitate lesion elevation. A circumferential incision was first made 0.5 cm outside the lesion margin with a Golden knife, followed by submucosal dissection. During dissection, marked submucosal fibrosis was encountered, with interspersed yellow granular deposits histopathologically consistent with schistosome eggs, corroborated by the patient's clinical history (Figure 3). The dissection was continued using an IT knife, enabling meticulous separation along the submucosal plane until complete *en bloc* resection was achieved (Figure 4). Intraoperatively, minor pinpoint bleeding foci were effectively managed with low-power electrocoagulation using hot biopsy forceps, achieving immediate hemostasis. Postprocedural wound inspection demonstrated satisfactory hemostasis without evidence of active hemorrhage. The total procedural duration was approximately 6 h. Postoperatively, the patient was given enhanced anti-infective therapy and fluid replacement. Enteral nutrition was initiated on postoperative day 3, and the patient was discharged uneventfully on January 12, 2026. No delayed bleeding or perforation has occurred since the operation.

**Figure 5:**
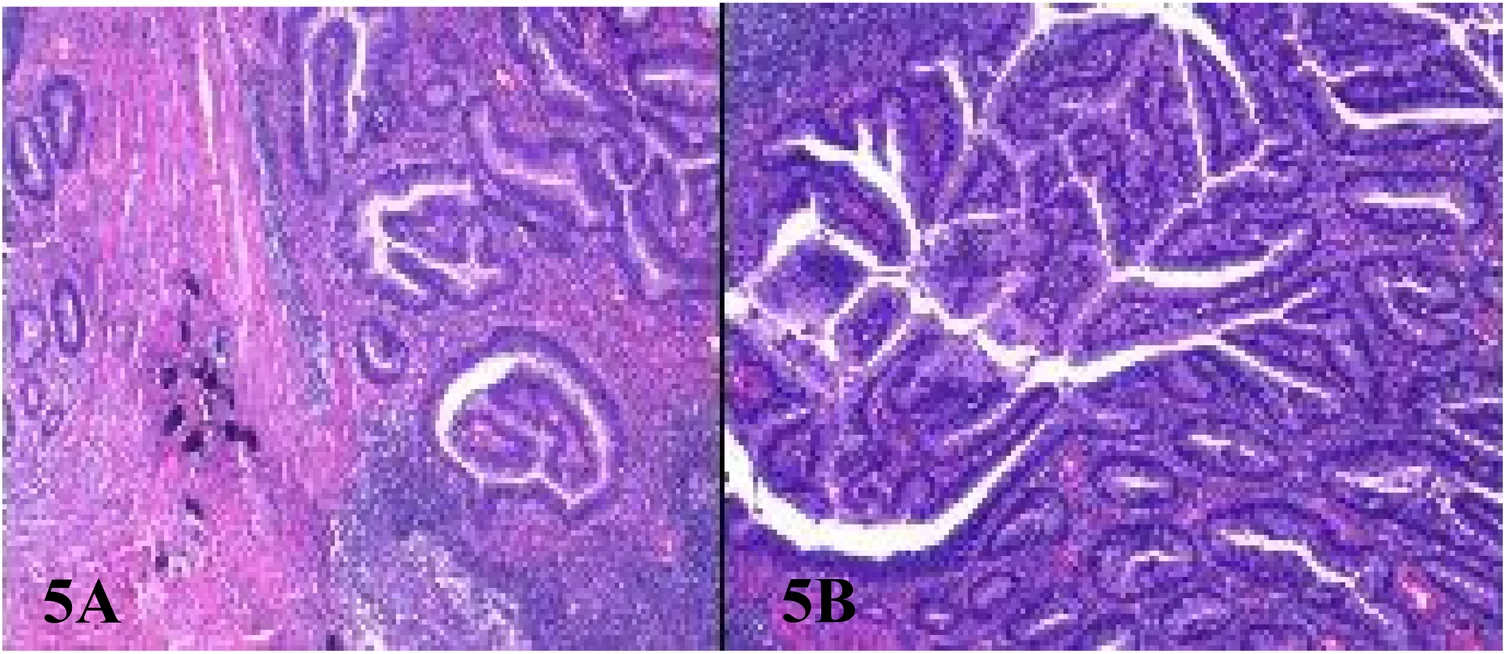
Postoperative pathology revealed a tubulovillous adenoma with high-grade intraepithelial neoplasia in some glands and focal carcinoma. The carcinoma had invaded the submucosa with an invasion depth of 660 um (measured from the muscularis mucosa). The resection margins were negative, and no tumor thrombus was found in the blood vessels. Old schistosome egg deposition was also noted.

The postoperative pathological examination revealed moderately differentiated invasive adenocarcinoma. The carcinoma invaded into the submucosa, with a depth of 660 um from the muscularis mucosae. The resection margins were negative, and no tumor thrombus was found in blood vessels or lymphatics. Tumor budding was graded as Grade 1. Additionally, schistosome egg deposition was identified within the tumor (Figure 5).

**Figure 6:**
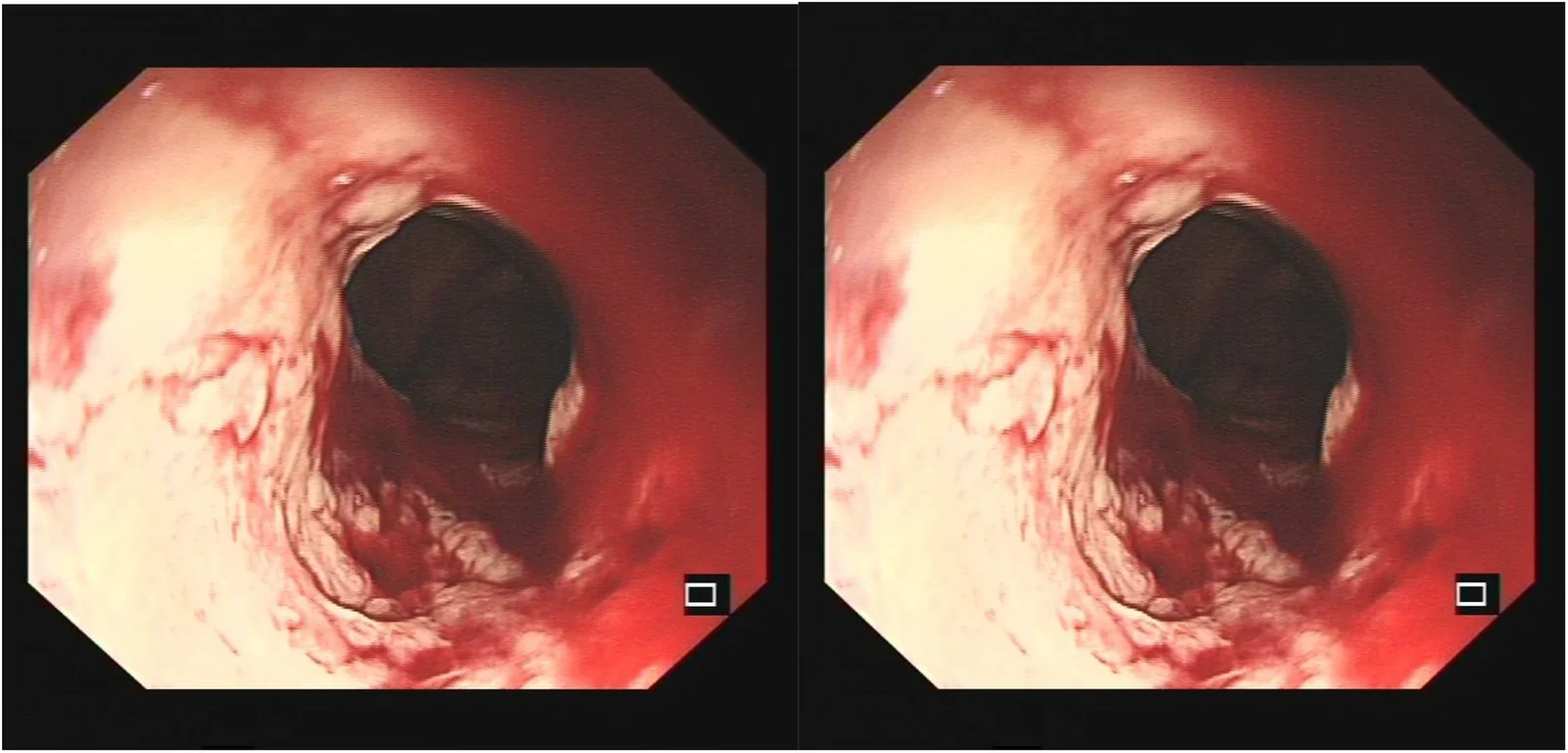
The patient was found to have a rectal stenosis located 2–8 cm from the anal verge.

Follow-up colonoscopy performed on April 9, 2026, demonstrated rectal stenosis extending from 2 to 8 cm from the anal verge, which was successfully dilated to 15 mm using a guidewire-directed balloon dilator. Subsequent diagnostic endoscopy traversed the stenotic segment without difficulty, revealing no evidence of neoplastic recurrence (Figure 6). During telephone follow-up on April 15, 2026, the patient expressed: “When I was first informed that I had a large circumferential lesion right next to the anal verge, I was extremely worried. The doctors told me that because of its size and location, removing it all at once with ESD would be very difficult, and I feared I might need major surgery that could affect my bowel function permanently. After the successful *en bloc* ESD procedure, I felt a huge weight lifted off my shoulders. I never imagined such a large and complex lesion could be completely removed in one piece without open surgery. I hope that in the future, patients with similar conditions can be fully informed about advanced endoscopic treatment options, so they do not have to face unnecessary anxiety about radical surgery. I will also strictly follow the recommended surveillance schedule to monitor for recurrence.” The patient additionally reported that etiological treatment for schistosomal hepatic cirrhosis had been deferred for personal and familial reasons, with only symptomatic management implemented.

**Figure 7:**
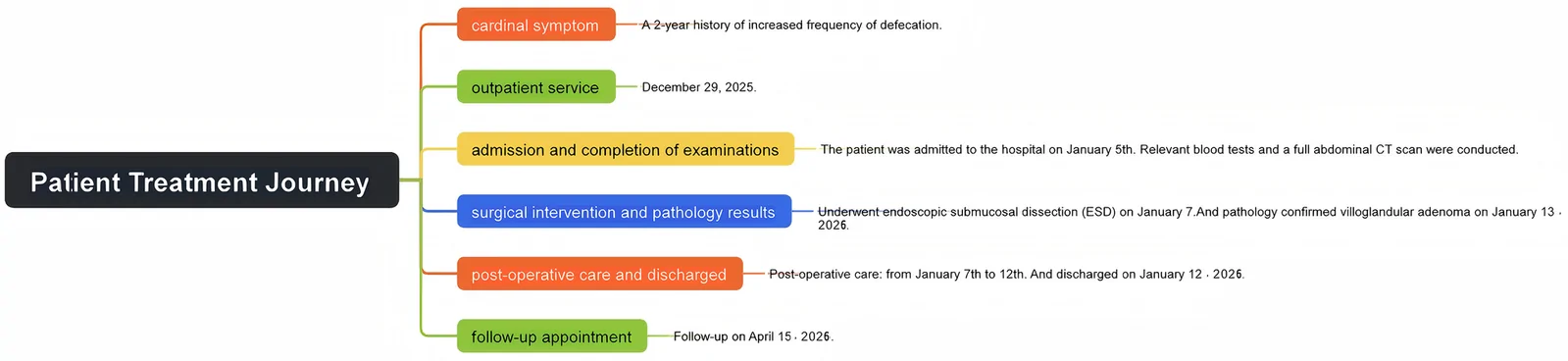
A timeline figure. Years not indicated in the figure are assumed to be 2026.

In summary, the patient had a two-year history of increased bowel movement frequency prior to visiting our outpatient clinic on December 29, 2025. He was admitted on January 5, 2026, and underwent relevant blood tests and CT examinations. After completing preoperative preparations, he underwent endoscopic submucosal dissection (ESD) on January 7. Postoperatively, he received broad-spectrum antibiotics and intravenous resuscitation therapy, and was discharged smoothly on January 12, 2026. Pathological results from January 13 revealed that the lesion was moderately differentiated adenocarcinoma. The first postoperative follow-up visit was conducted on April 15, 2026 (Figure 7).

## Discussion

This case illustrates a uniquely challenging clinical scenario involving a giant circumferential rectal LST with superficial submucosal invasion, further complicated by schistosomal colopathy-induced severe submucosal fibrosis The successful ESD of this lesion (maximum diameter >10 cm, adjacent to the anal verge) not only demonstrates the feasibility of ESD in managing high-difficulty rectal LSTs but also provides valuable clinical insights regarding the diagnosis and management of LSTs occurring in the context of chronic schistosomal infection.

A landmark Japanese study reported that submucosal invasion occurred in 29.4% of LSTs treated with ESD, of which 53.3% represented superficial submucosal invasions (6). The 2024 WEO International Consensus emphasizes that *en bloc* resection is the preferred strategy for rectal LSTs at high risk of superficial submucosal invasion, such as granular mixed-type lesions, to minimize local recurrence and prevent malignant progression. However, *en bloc* ESD resection for giant circumferential LSTs remains technically demanding (7), especially when lesions are situated immediately adjacent to the anal verge. The distal rectum presents unique anatomical challenges, including a narrow luminal diameter and relatively thin intestinal wall, which substantially elevate the risks of perforation and bleeding during dissection. In the present case, the lesion extended circumferentially from the anal verge to 8 cm proximally and the initial recommendation of surgical resection was abandoned due to the impossibility of anal preservation—highlighting the clinical value of ESD in preserving organ function for distal rectal lesions.

The presence of schistosomal colopathy further exacerbated the technical complexity of ESD in this case. Chronic schistosomiasis infection induces persistent intestinal inflammation, which precipitates progressive submucosal fibrosis and extensive schistosome egg deposition within the intestinal wall—findings directly observed intraoperatively as yellow granular deposits and severe tissue fibrosis, subsequently confirmed histopathologically. Fibrosis alters the normal submucosal anatomical plane, increases tissue stiffness, and hinders submucosal injection and dissection (8)—factors that are associated with higher ESD-related complication rates and incomplete resection rates. Nevertheless, by intraoperatively adapting our technique—switching to an IT knife upon encountering dense fibrosis and meticulously dissecting along residual normal submucosal planes—we achieved complete *en bloc* resection with histologically negative margins. This successful outcome underscores the critical importance of flexible instrument selection and proficient anatomical recognition during complex ESD procedures.

Postoperative pathology results provide critical implications for prognosis and follow-up. The lesion was diagnosed as villous tubular adenoma with high-grade intraepithelial neoplasia and focal moderately differentiated adenocarcinoma, invading the submucosa to a depth of 660 um. According to the WEO consensus, superficial submucosal invasion (≤1000 um) of rectal LSTs is associated with a low risk of lymph node metastasis, and *en bloc* resection with negative margins can achieve curative effects. Additionally, the absence of vascular/lymphatic tumor thrombi and low-grade tumor budding (Grade 1) further indicate a favorable prognosis.

Notwithstanding these favorable prognostic factors, the extensive schistosome egg deposition warrants particular clinical attention. Chronic schistosomal inflammation represents an established risk factor for colorectal mucosal dysplasia and carcinogenesis, potentially elevating the risk of metachronous polyp formation or subsequent neoplasia. Schistosomiasis remains highly endemic in Southeast Asia and is widely recognized as an independent risk factor for CRC development (9). Accumulating evidence demonstrates that schistosomiasis-associated colorectal cancer (SA-CRC) exhibits distinct epidemiological, histopathological, and clinical characteristics compared with non-schistosomiasis-related CRC. Genomic analyses have further elucidated the unique molecular landscape of SA-CRC, providing novel insights into its etiopathogenesis (10). However, conflicting evidence exists regarding prognostic implications: some studies indicate that schistosomiasis represents an independent adverse prognostic factor for patients with stage III–IV tumors and those with lymph node metastases, but not for patients with stage I–II disease or node-negative tumors (11).

Given the incompletely understood pathogenesis of SA-CRC, individualized postoperative surveillance is particularly critical. In accordance with current colorectal neoplasm surveillance guidelines (12), and tailored to this patient's unique clinical profile—giant circumferential anal verge lesion, malignant histopathology, and chronic schistosomal colopathy—we implemented a structured surveillance protocol. Follow-up is scheduled at 1, 3, 6, and 12 months post-ESD, followed by annual colonoscopic surveillance for a minimum of 5 years. Postoperative assessments focus on oncologic recurrence surveillance, wound healing evaluation, stricture monitoring, and anorectal functional assessment. Colonoscopic evaluations at 3 and 6 months will assess scar remodeling, exclude local residual or recurrent disease, and dynamically monitor chronic mucosal changes related to schistosomal infection. Serum tumor markers (CEA, CA19-9) will be monitored biannually. Pelvic imaging or endorectal ultrasound will be performed at 12 months to exclude deep local recurrence. Long-term surveillance prioritizes early detection of neoplastic recurrence, prevention of anal stricture, and preservation of anorectal function in the context of chronically inflamed mucosa.

This case also highlights the need for comprehensive preoperative evaluation in LST patients. Preoperative abdominal CT revealed schistosomal hepatic calcifications, which prompted further inquiry into the patient's schistosomiasis history—information that proved invaluable for anticipating intraoperative fibrosis and strategically planning the dissection approach. Routine screening for schistosomiasis should be considered in endemic areas, as preoperatively identifying such comorbidities can help clinicians optimize surgical plans and reduce unexpected technical challenges.

Several limitations inherent to this study warrant acknowledgment. First, as a single case report, the clinical conclusions cannot be universally extrapolated to all patients presenting with giant circumferential laterally spreading lesions near the anal verge. Second, the follow-up duration remains relatively limited, and long-term oncologic outcomes—including tumor recurrence risk and postoperative anorectal functional recovery—require ongoing longitudinal surveillance. Third, the absence of a control group receiving alternative resection modalities precludes statistical demonstration of the comparative superiority of *en bloc* ESD versus piecemeal resection or transanal minimally invasive surgery. Finally, this technically demanding ESD procedure is highly operator-dependent, requiring advanced endoscopic proficiency, which may limit its widespread implementation in resource-limited community hospital settings.

## Conclusion

In summary, ESD can be a safe and effective alternative to surgery for giant circumferential rectal LSTs adjacent to the anal verge, even in the presence of schistosomal colopathy-induced fibrosis. Successful outcomes rely on strict patient selection, comprehensive preoperative evaluation, flexible surgical techniques, and careful postoperative management. This case enriches the clinical experience of managing complex rectal LSTs and emphasizes the value of ESD in preserving anal function while achieving curative resection.

## Data Availability

The datasets presented in this study can be found in online repositories. The names of the repository/repositories and accession number(s) can be found in the article/Supplementary Material.
